# Distinct healing dynamics associated with xenograft selection in guided bone regeneration

**DOI:** 10.1038/s41598-026-54078-y

**Published:** 2026-06-22

**Authors:** Ynara Maria Gomes de Sousa, Mateus Torres-Silva, Martina Andreia Lage Nunes, Izabella Sol, João Paulo Schmitt Lopes, Talita Gracielen Lima da Silva, Marlus da Silva Pedrosa, Daniela Ponzoni

**Affiliations:** 1 Department of Diagnosis and Surgery, Araçatuba School of Dentistry, São Paulo State University (UNESP), Rua José Bonifácio, 1193, Araçatuba, 16015-050 SP Brazil; 2 Department of Orofacial Sciences, School of Dentistry, University of California, 513 Parnassus Avenue, Medical Sciences Building, Room S-511, San Francisco, CA USA

**Keywords:** Bone regeneration, Guided tissue regeneration, Bone substitutes, Collagen, Hydroxyapatites, Xenograft, Biotechnology, Materials science, Medical research, Stem cells

## Abstract

Guided bone regeneration relies on biomaterials that support bone formation while modulating the healing environment; however, materials with similar indications may exhibit different biological behaviors over time. This study compared the performance of two bovine-derived bone substitutes containing collagen using a rat calvarial critical-size defect model. Fifty-four Wistar rats were allocated to three groups: blood clot (control), Bio-Oss Collagen^®^, and Extra Graft XG13^®^. Standardized 5-mm defects were created and treated under guided bone regeneration conditions using a resorbable collagen membrane. Animals were euthanized at 7, 14, and 28 days. Mineralized tissue formation and microarchitecture were assessed by micro-CT, while histological, histomorphometric, inflammatory, angiogenic, and collagen organization analyses were performed to characterize the healing process. Both biomaterials supported tissue formation within the defect compared to the control. Extra Graft XG13^®^ was associated with higher mineralized tissue volume and volume fraction at 14 and 28 days, with more favorable micro-CT-derived microarchitectural parameters and reduced porosity. Early inflammatory and angiogenic responses were comparable between biomaterials, yet defects treated with Extra Graft XG13^®^ exhibited greater new bone formation over time. A reduction in mineralized tissue volume from 14 to 28 days in this group, accompanied by increased collagen organization, may suggest a possible transition toward remodeling; however, this interpretation should be considered with caution due to the absence of direct remodeling markers. In contrast, Bio-Oss Collagen^®^ showed a more gradual pattern of tissue formation and delayed collagen maturation. Overall, Extra Graft XG13^®^ was associated with greater mineralized tissue formation and a higher proportion of organized collagen fibers at later time points, indicating differences in the progression of mineralized tissue formation and matrix organization over time compared to Bio-Oss Collagen^®^. These findings should be interpreted within the limitations of the experimental model.

## Introduction

Guided bone regeneration (GBR) is a predictable approach for the reconstruction of bone defects prior to implant placement^1,2^. It is based on the principle of creating a protected environment that allows bone to regenerate while excluding soft tissue infiltration^2^. This is achieved through the combined use of barrier membranes and osteoconductive biomaterials^3^. In this setting, the graft material serves as a scaffold that guides cellular migration, vascular ingrowth, and new bone formation, making its biological behavior critical for successful healing^1–3^.

Xenogeneic bone substitutes of bovine origin are widely used due to their biocompatibility, osteoconductive properties, and structural similarity to human bone^4–6^. These materials provide a mineral framework that supports bone deposition and space maintenance during healing. Bio-Oss Collagen^®^ is extensively investigated and commonly used as a reference material in experimental and clinical studies^7–16^. In contrast, other commercially available xenografts with similar indications have been introduced with variations in composition and structure. Among these, Extra Graft XG13^®^ has been developed with a different proportion of organic and inorganic components, including a higher collagen content as reported by the manufacturer^17^, although its in vivo behavior remains less well characterized.^18^.

Variations in composition, including the proportion of hydroxyapatite and collagen, may influence aspects of the regenerative process, as suggested in previous experimental studies comparing collagen-based xenografts with different compositions^18^. Hydroxyapatite provides structural stability and acts as a mineral scaffold that supports bone ingrowth, while collagen contributes to material cohesion, improves handling, and may facilitate cellular adhesion and migration^4^. In addition, collagen has been associated with early biological events such as vascularization and matrix deposition^19^ Variations in the relative proportion of these components may influence the early phases of healing and the progression of bone regeneration; however, their specific effects may depend on multiple material properties^18^. However, whether these compositional differences translate into distinct patterns of bone regeneration over time remains unclear^19,20^.

Bone regeneration is a dynamic and time-dependent process involving sequential phases of inflammation, angiogenesis, matrix deposition, mineralization, and remodeling^21^. Each stage contributes to the formation and maturation of new bone tissue^21^. While many studies focus primarily on the final amount of bone formed, differences in the progression and timing of these events may directly affect tissue organization and quality, which are clinically relevant outcomes^3^. Preclinical models using critical-size defects in rat calvaria under standardized GBR conditions, including the use of resorbable collagen membranes, allow controlled evaluation of biomaterial performance^22^. In this model, the membrane preserves the defect space and prevents soft tissue invasion, enabling bone regeneration to occur. The combination of micro-computed tomography, histological, and histomorphometric analyses allows assessment of bone quantity, microarchitecture, and biological responses such as vascularization and collagen organization^22,23^.

The aim of this study was to compare the in vivo behavior of two bovine-derived bone substitutes with collagen, Bio-Oss Collagen^®^ and Extra Graft XG13^®^, in a rat calvarial critical-size defect model. The null hypothesis was that both biomaterials would present similar patterns of bone regeneration over time.

## Materials and methods

### Study design

**Fig. 1:**
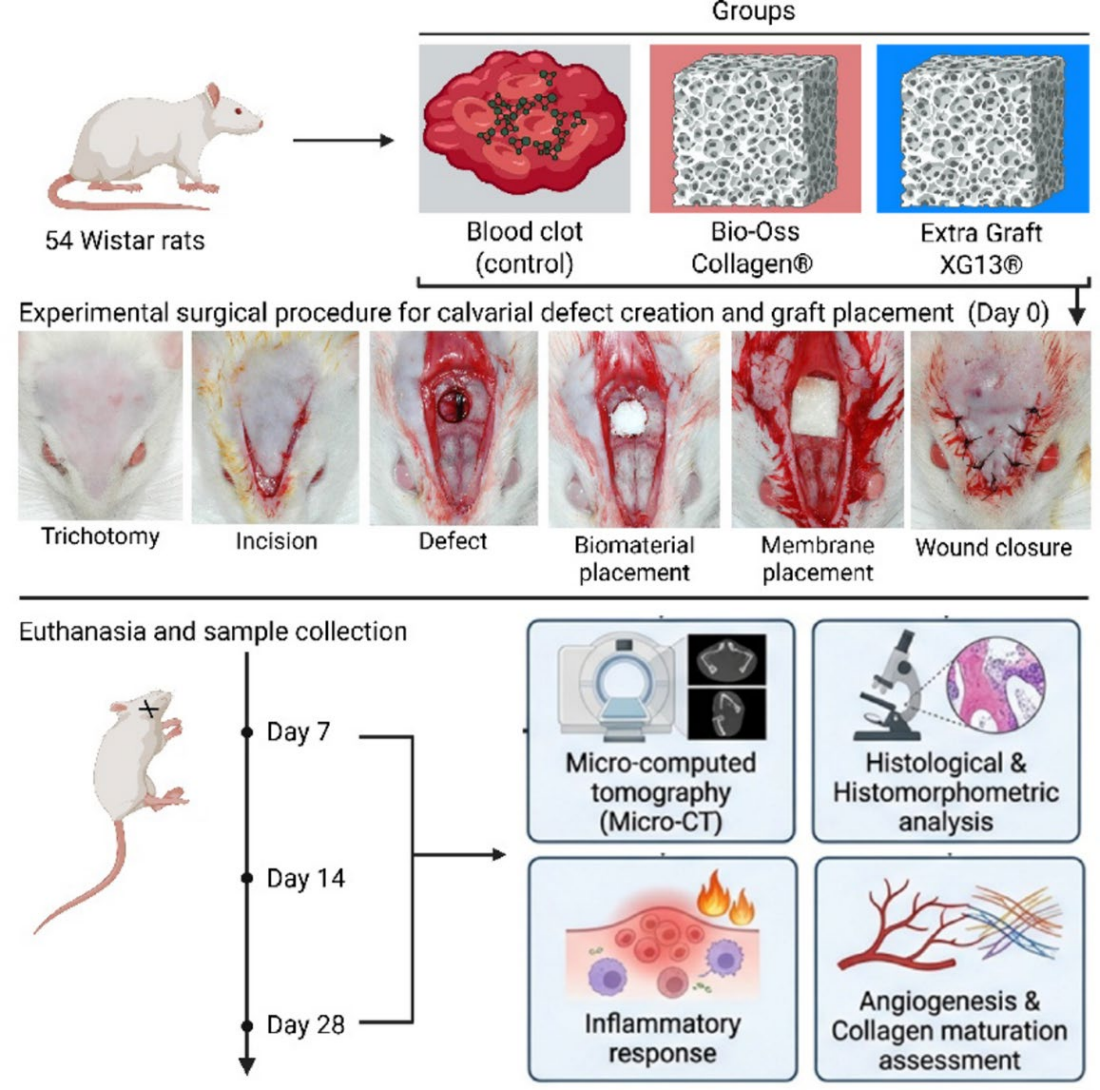
Experimental design and workflow of the study. Fifty-four Wistar rats were randomly allocated into three groups according to the treatment of the calvarial defect: blood clot (control), Bio-Oss Collagen^®^, and Extra Graft XG13^®^. A standardized 5-mm critical-size calvarial defect was created in all animals and treated under guided bone regeneration conditions using a resorbable collagen membrane (Day 0). Representative images of the experimental surgical procedure illustrate the main steps: trichotomy of the surgical area, incision, defect creation, biomaterial placement, membrane placement, and wound closure. Animals were euthanized at 7, 14, and 28 days for sample collection. Micro-computed tomography was performed at 14 and 28 days. Histological, histomorphometric, inflammatory, angiogenic, and collagen organization analyses were performed at all time points (7, 14, and 28 days). Created in BioRender. PEDROSA, M. (2026) https://BioRender.com/04tfgxi.

This in vivo experimental study was conducted in accordance with the ARRIVE guidelines and approved by the Animal Ethics Committee on the Use of Animals (CEUA), Araçatuba School of Dentistry, São Paulo State University (UNESP) (protocol no. 707/2024). All experimental procedures were performed in accordance with relevant guidelines and regulations. The experimental design is illustrated in Fig. 1. A critical-size defect model in rat calvaria was used to evaluate bone healing under standardized guided bone regeneration conditions.

### Animals and sample size

Fifty-four adult male Wistar rats (Rattus norvegicus), weighing 300–350 g, were obtained from the Central Animal Facility of the Araçatuba School of Dentistry, São Paulo State University (UNESP). Animals were housed under controlled temperature and light conditions, with free access to food and water. Sample size was determined based on previous studies,^24^ considering a significance level of 5% and a statistical power of 80%, resulting in six animals per group per time point (*n* = 6 per group).

### Experimental groups

Animals were randomly allocated into three groups according to the treatment of the calvarial defect using a computer-generated randomization sequence: control (blood clot), Bio-Oss Collagen^®^ (Geistlich, Switzerland), and Extra Graft XG13^®^ (Implacil, Brazil). Each group was further subdivided into three experimental periods (7, 14, and 28 days), with six animals per group at each time point (*n* = 6). All defects were treated under guided bone regeneration conditions using a resorbable collagen membrane (Green Membrane^®^, Regener, Brazil). The main characteristics of the biomaterials used in this study are summarized in Table 1.

**Table 1:** Characteristics of biomaterials used in the study.

Material	Manufacturer	Origin	Composition	Form	Function in study
Bio-Oss Collagen^®^	Geistlich, Switzerland	Bovine	~ 90% hydroxyapatite, ~ 10% collagen	Collagenated xenograft	Reference bone substitute
Extra Graft XG13^®^	Implacil, Brazil	Bovine	~ 75% hydroxyapatite, ~ 25% collagen*	Collagenated xenograft	Test bone substitute
Green Membrane^®^	Regener, Brazil	Bovine	Type I collagen	Resorbable membrane	Barrier for guided bone regeneration

* Composition based on manufacturer information.

### Surgical procedure

The surgical procedure is illustrated in Fig. 1. Animals were anesthetized with intramuscular ketamine (90 mg/kg) and xylazine (10 mg/kg). After trichotomy and antisepsis of the frontoparietal region, a linear incision was performed followed by flap elevation. A standardized 5-mm diameter bicortical defect was created in the center of the calvaria using a trephine bur under irrigation. Care was taken to preserve the integrity of the dura mater during defect creation. The periosteum was elevated during flap reflection and repositioned during closure, without removal. The defect size was considered critical, as spontaneous bone regeneration does not occur during the experimental period. Defects were filled according to group allocation.

The volume of biomaterial was standardized based on the defect dimensions. Considering the cylindrical geometry of the 5-mm defect, the volume was calculated using the formula V = πr^2^h, where V represents the volume of the defect, r corresponds to the defect radius (2.5 mm), and h to the thickness of the calvarial bone. Based on an average thickness of approximately 1.0 mm for adult Wistar rat calvaria, the calculated volume was approximately 19.6 mm^3^ per defect. This value was used as a reference to standardize the amount of biomaterial inserted in all experimental groups. This approach ensured consistent biomaterial volume across all defects. Due to the particulate nature of the biomaterials and the absence of rigid containment, slight variations in the contour of the grafted area may be observed in representative images, although the volume of material used was consistent across defects.

All defects were covered with a resorbable collagen membrane, and soft tissues were repositioned and sutured. The resorbable collagen membrane also contributed to stabilization of the graft material and prevention of displacement into the defect site. Animals received 24,000 IU/kg of Pentabiotic (Fort Dodge Animal Health Ltd., São Paulo, Brazil) and 50 to 100 mg/kg of Sodium Dipyrone (Ariston Chemical and Pharmaceutical Industries Ltd., São Paulo, Brazil) therapy after surgery and were monitored daily.

Euthanasia was performed at 7, 14, and 28 days by intraperitoneal administration of a triple overdose of anesthetic agents consisting of ketamine (270 mg/kg; Vetaset^®^, Fort Dodge Animal Health Ltd., Brazil) and xylazine (30 mg/kg; Dopaser^®^, Calier, Brazil). Death was confirmed by the absence of heartbeat and respiratory movement prior to specimen collection. Calvarial specimens were harvested and fixed in 10% buffered formalin.

### Inflammatory response and angiogenesis

Inflammatory cells and blood vessels were quantified using a point-counting method. Three regions of the defect (left margin, central region, and right margin) were analyzed at 100× magnification using a grid containing 130 points. Structures intersecting the grid points were counted. Histological, histomorphometric, inflammatory, and angiogenic analyses were performed by an examiner blinded to group allocation.

### Histological and histomorphometric analysis

Specimens were decalcified in EDTA, processed, and embedded in paraffin. Sections were obtained and stained with hematoxylin and eosin. Qualitative analysis assessed tissue organization, new bone formation, connective tissue, inflammatory infiltrate, and presence of biomaterial. New bone formation was defined based on morphological criteria, including osteoid matrix deposition and the presence of osteoblast-like cells, in accordance with established histological descriptions of early bone healing. Histomorphometric analysis was performed using digital images analyzed with ImageJ software. New bone formation was expressed as percentage of the original sample area.

### Micro-computed tomography (micro-CT)

Micro-computed tomography analysis was performed at 14 and 28 days to evaluate mineralized tissue formation and microarchitecture, as early time points predominantly consist of non-mineralized tissue. Samples were scanned using a high-resolution microtomograph (Skyscan 1172, Bruker, Belgium) with a voxel size of 11.87 μm, 50 kVp, 0.5 mm aluminum filter, and 0.6° rotation step. A standardized region of interest (ROI) corresponding to the original 5-mm diameter defect was defined based on anatomical landmarks and consistent alignment across samples. After scanning, images were reconstructed using NRecon software (Bruker, Belgium) and subsequently processed using DataViewer (Bruker, Belgium) for alignment and determination of the volume of interest (VOI). For computational purposes, a bounding box of approximately 5 × 5 mm was used to delimit the ROI during image processing, corresponding to the original defect and defined based on standardized anatomical alignment in the sagittal, coronal, and axial planes. Morphometric analysis was performed using CTAn software (Bruker, Belgium). The following parameters were evaluated: mineralized tissue volume (BV), mineralized tissue volume fraction (BV/TV**)**, trabecular number (Tb.N), trabecular thickness (Tb.Th), trabecular separation (Tb.Sp), and total porosity (Po.Tot). Segmentation was performed using a fixed global grayscale threshold applied consistently across all samples; however, due to the similar radiopacity of newly formed bone and residual xenograft particles, reliable differentiation between these components was not possible. Therefore, the reported parameters represent total mineralized tissue within the defect, reflecting the combined contribution of newly formed bone and residual biomaterial.

### Collagen maturation (Picrosirius Red)

Collagen organization was evaluated using Picrosirius Red staining under polarized light microscopy at 400× magnification. Collagen fibers were classified based on birefringence as immature (green spectrum), intermediate (yellow spectrum), or mature (red spectrum). Quantification was performed as percentage of total collagen area. Analysis was conducted within the defect region using standardized fields of view, and measurements reflect total collagen content within the analyzed area without distinction between newly synthesized collagen and residual biomaterial- or membrane-derived collagen.

### Statistical analysis

Statistical analysis was performed using GraphPad Prism software (version 11.0, GraphPad Software Inc., San Diego, CA, USA). Data normality was assessed using the Shapiro–Wilk test. Two-way analysis of variance (ANOVA) was applied considering experimental group and time as independent factors, followed by Tukey post hoc test for multiple comparisons. Main effects (group and time) and their interaction (group × time) were evaluated, and effect sizes were estimated using partial eta-squared (η^2^p). Post hoc comparisons were primarily performed between groups within the same time point, unless otherwise specified. All data are presented as mean ± standard deviation, and the level of significance was set at *p* < 0.05. Statistical significance is indicated in the figures using symbols defined in the corresponding figure legends. Exact p-values and pairwise comparisons are reported in the figure legends where applicable.

## Results

Two-way ANOVA revealed significant effects of group and time for the evaluated parameters, with significant group × time interactions observed for mineralized tissue formation, histomorphometric outcomes, inflammatory response, and collagen organization (*p* < 0.05), while angiogenesis and selected microarchitectural parameters did not show significant interaction effects.

### Greater mineralized tissue formation in defects treated with Extra Graft XG13^®^ compared to Bio-Oss Collagen^®^

**Fig. 2:**
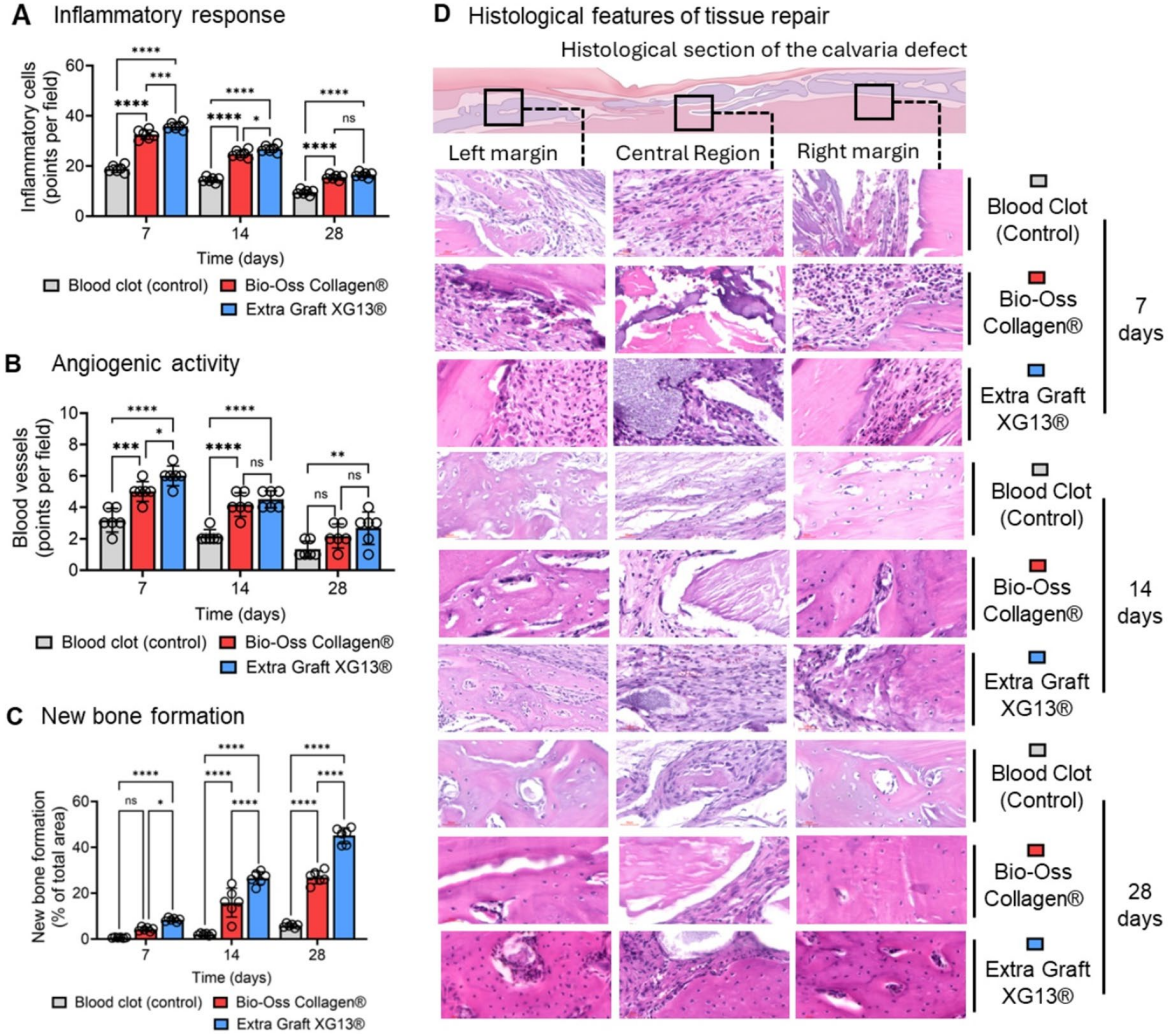
Biological response, angiogenesis, new bone formation, and histological features. A standardized 5-mm calvarial defect was created and treated under guided bone regeneration conditions using a resorbable collagen membrane. Bone healing was evaluated at 7, 14, and 28 days using histomorphometric and histological analyses. (**A**) Inflammatory response expressed as inflammatory cells (points), quantified using a point-counting method with a 130-point grid at 100x magnification in three standardized regions of the defect (left margin, central region, and right margin). (**B**) Angiogenic activity expressed as blood vessels (points), quantified using the same point-counting method and regions of interest as described for inflammatory cells. (**C**) Histomorphometric analysis of new bone formation within the defect area, measured by calculating the area of newly formed tissue relative to the original defect region using image analysis software. (**D**) Representative histological sections of the calvarial defect at 7, 14, and 28 days, illustrating the left margin, central region, and right margin for each experimental group. Images demonstrate the spatial distribution of newly formed bone, connective tissue, and residual biomaterial within the defect. At early time points, newly formed tissue was identified based on histological features consistent with early osteoid deposition and osteogenic activity, which may not represent fully mineralized bone. Data are presented as mean ± standard deviation (*n* = 6 per group per time point). Statistical analysis was performed using two-way ANOVA followed by Tukey post hoc test. Statistical significance (asterisks) indicates differences between groups within the same time point. *p < 0.05, **p < 0.01, ***p < 0.001, ****p < 0.0001; ns = not significant.

To evaluate the biological response over time and its relationship with mineralized tissue formation, inflammatory response, angiogenic activity, and new bone formation were analyzed at 7, 14, and 28 days (Fig. 2).

The inflammatory response was higher in the biomaterial groups compared to the control at early time points (Fig. 2A). At 7 days, both Bio-Oss Collagen^®^ and Extra Graft XG13^®^ groups showed significantly increased inflammatory cell counts compared to the control (*p* < 0.0001), with no significant difference between the biomaterials. A similar pattern was observed at 14 days, followed by a reduction in inflammatory cells over time in all groups (*p* < 0.001). At 28 days, inflammatory levels decreased further but remained higher in the biomaterial groups compared to the control. Two-way ANOVA revealed significant effects of group (η²*p* = 0.96) and time (η²*p* = 0.95), with a significant group × time interaction (η²*p* = 0.67), indicating a strong influence of both factors on the inflammatory response.

Angiogenic activity followed a comparable trend (Fig. 2B). At 7 days, both biomaterial groups exhibited significantly higher vessel counts compared to the control (*p* ≤ 0.002), with no significant differences between Bio-Oss Collagen^®^ and Extra Graft XG13^®^. This pattern persisted at 14 days (*p* < 0.001). At 28 days, vascularization decreased in all groups but remained higher in the biomaterial-treated defects compared to the control. Two-way ANOVA revealed significant effects of group (η²*p* = 0.75) and time (η²*p* = 0.68); however, no significant group × time interaction was observed (η²*p* = 0.17).

Despite the similar inflammatory and angiogenic responses, differences in bone formation were observed (Fig. 2C). New bone formation increased over time in all groups (*p* < 0.05). The control group consistently presented the lowest values at all time points. Bio-Oss Collagen^®^ showed intermediate values, with gradual bone formation over time. In contrast, defects treated with Extra Graft XG13^®^ exhibited significantly greater new bone formation at both 14 and 28 days compared to Bio-Oss Collagen^®^ and control groups (*p* < 0.05). Histological analysis supported these findings (Fig. 2D). At 7 days, bone formation was restricted to the margins. At 14 days, increased organization of connective tissue and initial bone formation were observed in all groups, with more evident bone deposition in the biomaterial-treated defects. At 28 days, more advanced tissue organization and bone formation were observed, with defects treated with Extra Graft XG13^®^ showing a more extensive distribution of newly formed bone within the defect compared to Bio-Oss Collagen^®^ and control groups. Two-way ANOVA revealed significant effects of group (η²*p* = 0.92) and time (η²*p* = 0.94), with a significant group × time interaction (η²*p* = 0.81), indicating that the progression of new bone formation differed between the experimental groups.

Overall, while inflammatory and angiogenic responses were comparable between biomaterials, defects treated with Extra Graft XG13^®^ demonstrated greater mineralized tissue formation, indicating differences in healing progression.

### Time-dependent mineralized tissue formation and microarchitecture assessed by micro-CT, with greater mineralized tissue formation in Extra Graft XG13^®^

**Fig. 3:**
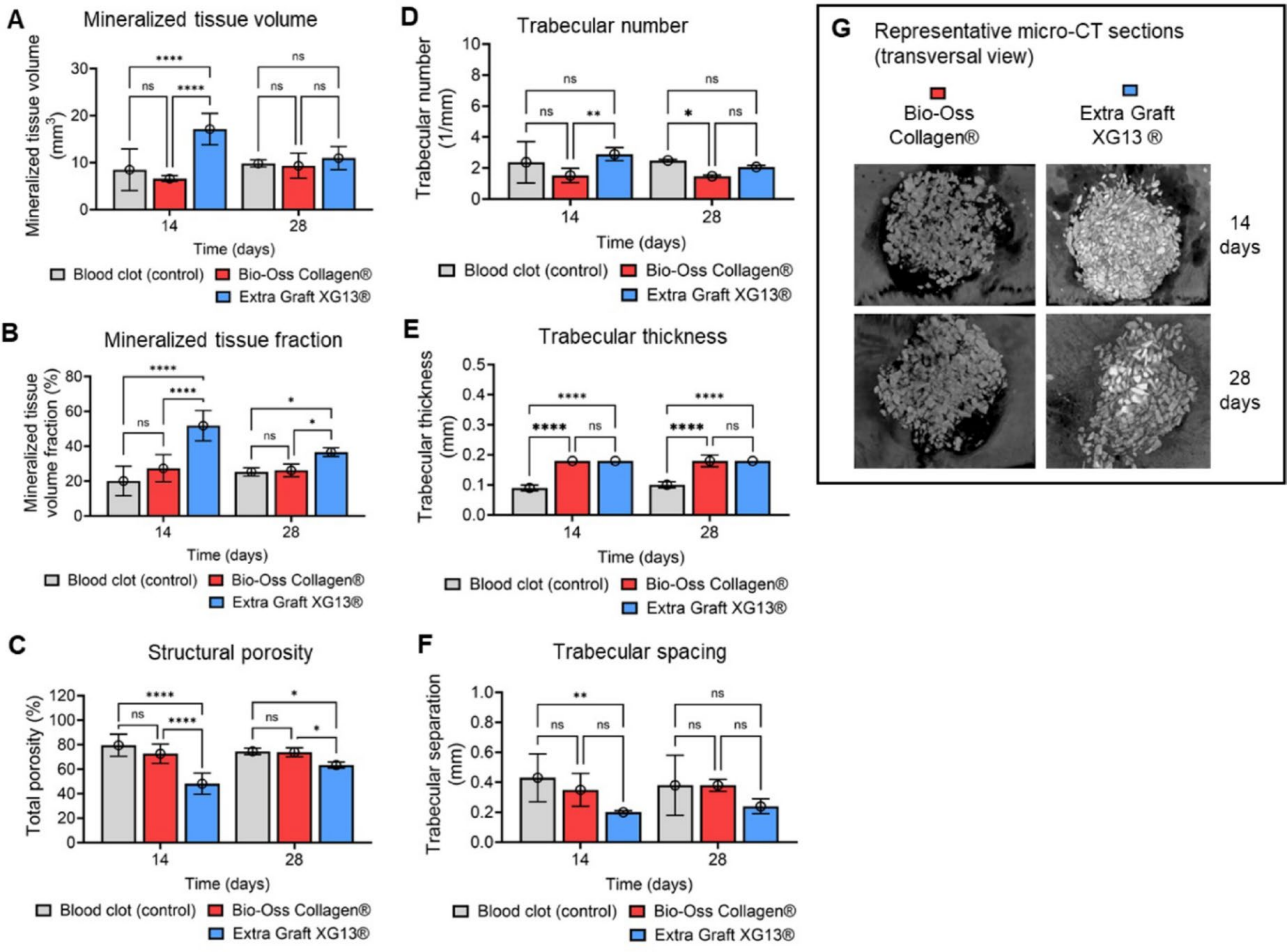
Micro-computed tomography analysis of mineralized tissue formation and trabecular microarchitecture. Mineralized tissue formation was evaluated at 14 and 28 days using micro-CT allowing quantitative assessment of mineralized tissue volume and microarchitectural parameters within the defect region. The analyzed region corresponds to the original defect area (ROI), defined using standardized anatomical landmarks. (**A**) Mineralized tissue volume (mm^3^), representing the total amount of mineralized tissue (including residual biomaterial and newly formed mineralized tissue) within the sample. (**B**) Mineralized tissue volume fraction (% of total volume), indicating the proportion of mineralized tissue relative to the total tissue volume within the defect. (**C**) Structural porosity (% of volume), reflecting the proportion of void spaces within the mineralized structure. (**D**) Trabecular density (trabecular number, 1/mm), representing the number of trabeculae per unit length. (**E**) Trabecular thickness (mm), indicating the average thickness of trabeculae. (**F**) Trabecular spacing (mm), representing the distance between trabeculae. (**G**) Representative micro-computed tomography cross-sectional images of the calvarial defect for Bio-Oss Collagen^®^ and Extra Graft XG13^®^ at 14 and 28 days, illustrating the distribution of mineralized components within the defect region. Data are presented as mean ± standard deviation (*n* = 6 per group per time point). Statistical analysis was performed using two-way ANOVA followed by Tukey post hoc test. Statistical significance (asterisks) indicates differences between groups within the same time point. *p < 0.05, **p < 0.01, ***p < 0.001, ****p < 0.0001; ns = not significant.

Micro-computed tomography analyses were performed at 14 and 28 days to capture bone formation during the active formation phase and its subsequent progression (Fig. 3). At 14 days, defects treated with Extra Graft XG13^®^ exhibited significantly higher mineralized tissue volume compared to both the control and Bio-Oss Collagen^®^ groups (*p* < 0.0001 and *p* < 0.01, respectively) (Fig. 3A). A similar pattern was observed for mineralized tissue volume fraction, with Extra Graft XG13^®^ showing greater values than both groups (*p* < 0.0001) (Fig. 3B). These findings indicate increased mineralized tissue formation at earlier stages in defects treated with Extra Graft XG13^®^. A significant group × time interaction was observed for mineralized tissue volume (*p* = 0.0007, η²*p* = 0.38) and mineralized tissue volume fraction (*p* = 0.0013, η²*p* = 0.36), indicating differences in temporal progression between biomaterials, with time showing a strong effect on both parameters.

Regarding microarchitectural parameters, structural porosity was significantly lower in the Extra Graft XG13^®^ group compared to the control and Bio-Oss Collagen^®^ groups at 14 days (*p* < 0.0001), indicating a denser tissue structure (Fig. 3C). Extra Graft XG13^®^ also demonstrated higher trabecular density compared to Bio-Oss Collagen^®^ (*p* < 0.01) (Fig. 3D). In addition, both biomaterial groups exhibited greater trabecular thickness compared to the control (*p* < 0.0001) (Fig. 3E). Trabecular spacing was reduced in the Extra Graft XG13^®^ group compared to the control (*p* < 0.05) (Fig. 3F).

At 28 days, mineralized tissue volume and mineralized tissue volume fraction remained higher in the Extra Graft XG13^®^ group compared to Bio-Oss Collagen^®^ and control groups (*p* < 0.05) (Figs. 3A, B). A reduction in mineralized tissue volume between 14 and 28 days was observed in the Extra Graft XG13^®^ group; however, this finding should be interpreted with caution, as micro-computed tomography does not allow reliable differentiation between newly formed bone and residual biomaterial. Structural porosity remained lower in the Extra Graft XG13^®^ group compared to both Bio-Oss Collagen^®^ and control groups (*p* < 0.05) (Fig. 3C). Differences in trabecular density between biomaterials were reduced, although the control group presented higher values than Bio-Oss Collagen^®^ (*p* < 0.05) (Fig. 3D). Trabecular thickness remained significantly higher in both biomaterial groups compared to the control (*p* < 0.0001) (Fig. 3E). It should be noted that micro-computed tomography measurements reflect total mineralized tissue within the defect and do not allow differentiation between newly formed bone and residual biomaterial.

Representative micro-computed tomography cross-sectional images support the quantitative findings, demonstrating greater filling of the defect and a more compact structure in defects treated with Extra Graft XG13^®^, particularly at 14 days (Fig. 3G).

Overall, micro-computed tomography analysis revealed that defects treated with Extra Graft XG13^®^ presented greater mineralized tissue formation and a more favorable microarchitecture at earlier time points, while differences between biomaterials became less pronounced over time.

### Time-dependent collagen organization showing differences between Extra Graft XG13^®^ and Bio-Oss Collagen^®^

**Fig. 4:**
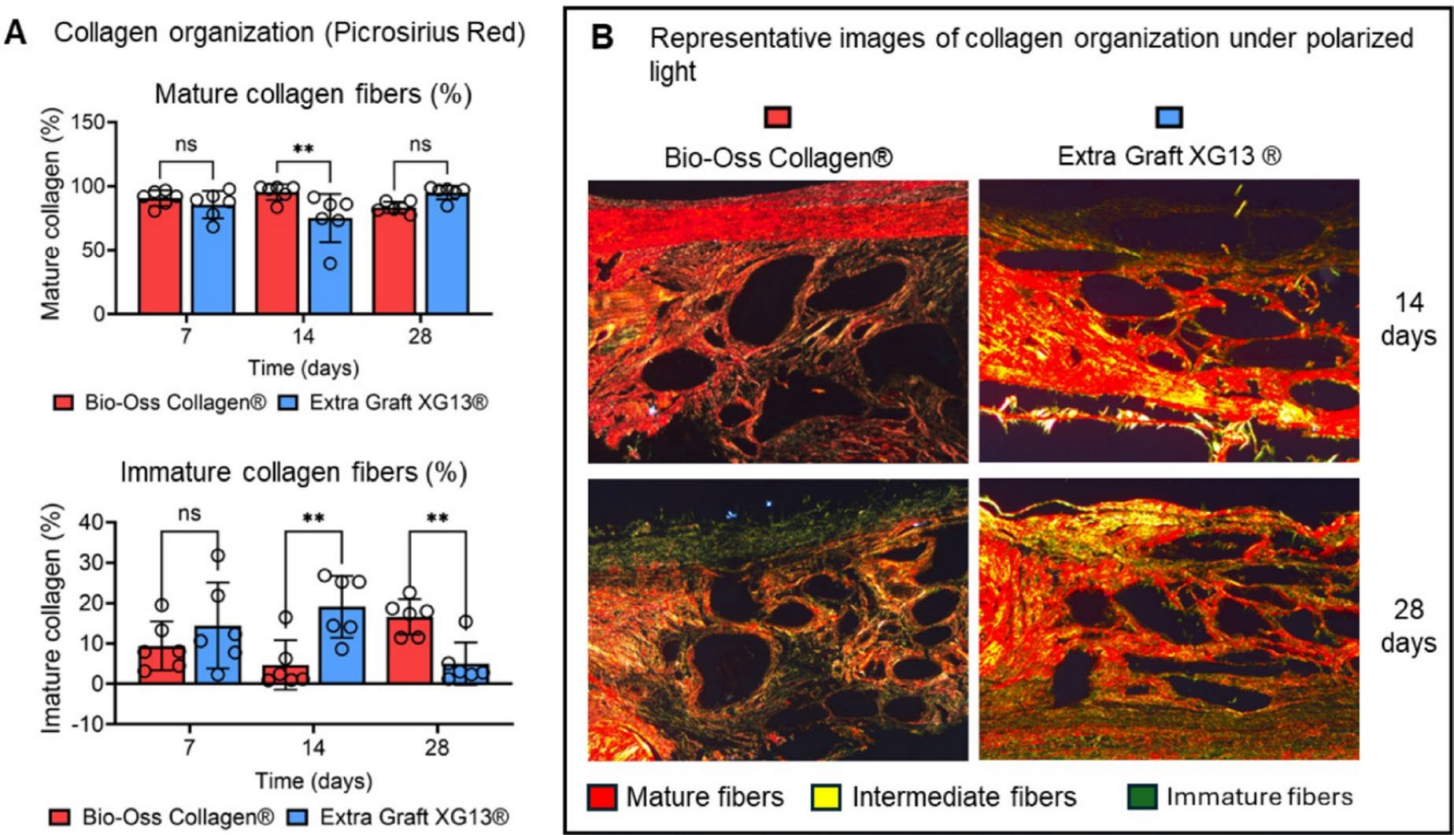
Collagen organization during bone formation. Collagen maturation was evaluated at 7, 14, and 28 days using Picrosirius Red staining under polarized light microscopy, allowing differentiation between immature and mature collagen fibers based on birefringence. Collagen birefringence reflects total collagen content within the analyzed area and does not distinguish between newly formed and biomaterial-derived collagen. (**A**) Quantitative analysis of collagen organization. Mature collagen fibers (upper graph) and immature collagen fibers (lower graph), both expressed as percentage of total collagen area. (**B**) Representative images of collagen fiber organization under polarized light for Bio-Oss Collagen^®^ and Extra Graft XG13^®^ Upper panels correspond to 14 days and lower panels to 28 days. Mature (red), intermediate (yellow), and immature (green) birefringence indicate different stages of collagen fiber maturation within the defect area. Data are presented as mean ± standard deviation (*n* = 6 per group per time point). Statistical analysis was performed using two-way ANOVA followed by Tukey post hoc test. Statistical significance (asterisks) indicates differences between groups within the same time point. *p < 0.05, **p < 0.01, ***p < 0.001; ns = not significant.

Collagen maturation was evaluated at 7, 14, and 28 days using Picrosirius Red staining under polarized light, allowing differentiation between immature and mature collagen fibers (Fig. 4). At 7 days, both groups predominantly exhibited immature collagen fibers, with no significant differences between Bio-Oss Collagen and Extra Graft XG13^®^ (Fig. 4A). Significant group × time interactions were observed for both mature (η²*p* = 0.34) and immature collagen fibers (η²*p* = 0.41), indicating differences in collagen organization over time between groups.

At 14 days, differences in collagen organization became evident. The Extra Graft XG13^®^ group showed a higher proportion of immature collagen fibers compared to Bio-Oss Collagen (*p* < 0.05), indicating a predominance of less organized collagen at this time point. Conversely, Bio-Oss Collagen presented a greater proportion of mature fibers at this time point (*p* < 0.05).

At 28 days, this pattern shifted. The Extra Graft XG13^®^ group exhibited a significantly higher proportion of mature collagen fibers compared to Bio-Oss Collagen (*p* < 0.01), while the proportion of immature fibers decreased accordingly (Fig. 4A), indicating a greater degree of collagen organization at this time point.

Representative polarized light images support these findings (Fig. 4B). At 14 days, both groups showed predominance of green birefringence, indicating immature collagen, with more extensive collagen deposition observed in the Extra Graft XG13^®^ group. At 28 days, an increase in red and yellow birefringence was observed, particularly in the Extra Graft XG13^®^ group, reflecting more advanced collagen maturation and tissue organization.

Overall, collagen maturation analysis indicates a shift from immature to mature collagen over time in both groups, with defects treated with Extra Graft XG13^®^ demonstrating a more advanced maturation pattern at later time points.

## Discussion

Guided bone regeneration relies on biomaterials that not only provide structural support but also influence the biological processes underlying tissue repair^1–3.^ Increasing attention has been directed toward understanding how different biomaterials affect the temporal dynamics of healing, rather than solely the final amount of regenerated tissue^1–3.^ This study evaluated the in vivo behavior of two bovine-derived bone substitutes with collagen under standardized guided bone regeneration conditions. Within the limitations of the experimental model, both biomaterials supported mineralized tissue formation; however, differences in time-dependent healing patterns were observed. Despite comparable early inflammatory and angiogenic responses, defects treated with Extra Graft XG13^®^ exhibited greater mineralized tissue formation at intermediate time points, whereas Bio-Oss Collagen^®^ demonstrated a more gradual pattern of tissue formation over time. At early time points, the identification of new bone formation should be interpreted as reflecting initial osteoid deposition and early matrix formation rather than fully mineralized bone tissue. These findings indicate differences in the progression of healing, although the underlying biological mechanisms cannot be definitively established based on the present data.

The early phase of healing was characterized by increased inflammatory response and angiogenesis in the biomaterial groups compared to the control (Fig. 2). However, no statistically significant differences were observed between Bio-Oss Collagen^®^ and Extra Graft XG13^®^ at these early time points, indicating that both materials established a comparable initial biological environment. These findings suggest that inflammatory and angiogenic responses alone do not account for the differences observed in mineralized tissue formation between the biomaterials. Angiogenesis plays a critical role in this process by supplying oxygen, nutrients, and progenitor cells required for tissue formation^21^. Within the limitations of the present data, these processes should be interpreted as part of the overall healing environment rather than as determinants of intergroup differences.

Micro-computed tomography analysis demonstrated that Extra Graft XG13^®^ promoted greater mineralized tissue formation and improved microarchitecture at earlier stages (Fig. 3). Higher mineralized tissue volume and mineralized tissue volume fraction, along with more favorable trabecular parameters, indicate a denser mineralized structure with more favorable micro-CT-derived parameters. Micro-computed tomography analysis does not allow differentiation between newly formed bone and residual graft material, and therefore the reported parameters reflect total mineralized tissue rather than exclusively newly formed bone. Notably, these differences were more pronounced at 14 days and became less distinct at 28 days, indicating a time-dependent effect. These findings highlight that differences between biomaterials may be more evident at early stages of healing, which are often underrepresented in preclinical studies. In contrast, Bio-Oss Collagen^®^ showed a slower but continuous increase in bone formation, consistent with its well-documented structural stability and slow resorption profile^4^. These findings were supported by histomorphometry analysis, which confirmed greater new bone formation in the Extra Graft XG13^®^ group at 14 and 28 days (Fig. 2).

An important observation was the reduction in mineralized tissue volume in the Extra Graft XG13^®^ group between 14 and 28 days (Fig. 3), accompanied by increased collagen maturation and histological features consistent with tissue reorganization (Fig. 4). This pattern may suggest a possible earlier transition from bone formation toward remodeling; however, this interpretation should be considered with caution. Bone healing is a dynamic process in which initial matrix deposition is followed by resorption and replacement by more organized tissue^21.^ In the absence of direct remodeling markers, such as osteoclast activity, TRAP staining, or lamellar bone assessment, the present findings do not allow definitive identification of remodeling processes. The shift from immature to mature collagen fibers observed in this study, including the transition from green to yellow and red birefringence under polarized light, should be interpreted as indicative of changes in collagen organization rather than definitive evidence of newly formed bone matrix maturation. It should be noted that Picrosirius Red staining does not allow differentiation between newly synthesized collagen and collagen derived from residual biomaterial or the collagen membrane, which may influence the interpretation of these findings, particularly at early time points. Given that Extra Graft XG13^®^ contains a higher proportion of collagen, the increased collagen signal observed may partially reflect the presence and remodeling of biomaterial-derived collagen in addition to newly formed extracellular matrix.

The findings of the present study can be compared with the experimental work by Gehrke et al., who evaluated collagen-based xenografts with different compositions in a rabbit calvarial model^18^. In that study, a biomaterial containing a higher proportion of collagen was associated with greater bone formation at later healing stages. While this observation is in line with the differences observed in the present study, such comparisons should be interpreted with caution, as no physicochemical characterization was performed and the underlying mechanisms cannot be directly established. In addition, differences in animal models and experimental time points limit direct comparison. Notably, in the present study, differences were observed at earlier time points, suggesting that variations in biomaterial behavior may influence the initial phases of healing.

Within the limitations of the present experimental model, these findings indicate differences in healing dynamics between biomaterials; however, their direct clinical relevance remains uncertain and should be interpreted with caution. Materials that promote faster early mineralized tissue formation may warrant further investigation in future preclinical or translational studies, particularly in studies evaluating healing progression; however, this interpretation should be regarded as hypothesis-generating. In clinical practice, healing time is a relevant factor in procedures such as ridge preservation and staged implant placement, where defined healing intervals are often required prior to implant insertion^1^. The present study was conducted in a short-term, non-load-bearing rat calvarial model and did not evaluate implant placement, mechanical properties, long-term volume stability, or functional outcomes. Therefore, extrapolation of these findings to clinical practice should be made with caution. Conversely, biomaterials with slower resorption and more sustained structural support, such as Bio-Oss Collagen^®^, may warrant further investigation in experimental models evaluating volume stability over time.

Some limitations should be considered. A key limitation of this study is the absence of independent physicochemical characterization of the biomaterials, which precludes direct correlation between specific material properties and the observed biological outcomes. In addition, compositional information was based on manufacturer-provided data, which may limit detailed interpretation of structure–function relationships. The experimental period was limited to 28 days, which does not allow direct evaluation of long-term remodeling outcomes or the stability of the regenerated tissue over time. While the selected time points capture early and intermediate phases of healing, including matrix deposition and mineralized tissue formation, they do not reflect later stages of tissue maturation and remodeling. In addition, molecular and biomechanical analyses were not performed, which could provide further insight into the biological mechanisms and functional properties associated with the observed differences. Picrosirius Red staining does not differentiate between residual biomaterial-derived collagen and newly synthesized collagen matrix, which may influence the interpretation of collagen organization patterns. Furthermore, physicochemical properties of the biomaterials, such as porosity, surface characteristics, and degradation behavior, were not directly evaluated, limiting correlation between material structure and biological response. Future studies incorporating gene expression, protein markers, and mechanical testing would be valuable to better characterize the regenerative process. Finally, although the rat calvarial model is widely used and well established, it does not fully reproduce the clinical conditions found in humans, particularly regarding biomechanical loading and defect complexity^22^. In this context, the non-load-bearing nature of the model may influence the observed healing dynamics. Therefore, further investigations using larger animal models and clinical studies are necessary to validate the translational relevance of these findings and to determine their applicability in different clinical scenarios.

Overall, both biomaterials demonstrated osteoconductive potential. However, Extra Graft XG13^®^ exhibited a more rapid and dynamic pattern of mineralized tissue formation, whereas Bio-Oss Collagen^®^ showed a slower and more sustained response. These findings indicate that biomaterials with similar indications may present different regeneration dynamics, which may inform future preclinical and translational investigations evaluating biomaterial performance in guided bone regeneration.

## Conclusion

Both bovine-derived bone substitutes demonstrated osteoconductive potential and supported bone healing in a rat calvarial critical-size defect model. However, distinct time-dependent healing patterns were observed. Extra Graft XG13^®^ was associated with greater mineralized tissue formation, improved microarchitectural parameters at earlier time points, and a higher proportion of organized collagen fibers compared to Bio-Oss Collagen^®^. Despite similar inflammatory and angiogenic responses, defects treated with Extra Graft XG13^®^ exhibited differences in the progression of mineralized tissue formation and matrix organization over time.

## Data Availability

All data supporting the findings of this study are available within the paper. Additional datasets generated and/or analyzed during the current study are available from the corresponding author on reasonable request.
